# Identification and attribute analysis of key stakeholders who influence multidrug-resistant tuberculosis prevention and control in China

**DOI:** 10.1186/s40249-021-00892-7

**Published:** 2021-08-12

**Authors:** Bin Chen, Hongdan Bao, Xinyi Chen, Kui Liu, Ying Peng, Wei Wang, Fei Wang, Jianmin Jiang, Biao Xu

**Affiliations:** 1 Department of Epidemiology, School of Public Health, Fudan University, Shanghai, China; 2 Zhejiang Provincial Center for Disease Control and Prevention, Hangzhou, Zhejiang China; 3 Medical Insurance Management Office, Zhejiang Hospital, Hangzhou, Zhejiang China; 4 Key Laboratory of Vaccine, Prevention and Control of Infectious Disease of Zhejiang Province, Hangzhou, Zhejiang, China; 5 Key Laboratory of Health Technology Assessment, National Health Commission of the People’s Republic of China (Fudan University), Shanghai, China

**Keywords:** Multidrug-resistant tuberculosis, Stakeholders, Prevention and control

## Abstract

**Background:**

There could be various stakeholders who influencing multidrug-resistant tuberculosis (MDR-TB) policy development and implementation, yet their attributes and roles remain unclear in practice. This study aimed to identify key stakeholders in the process of policy-making for MDR-TB control and prevention and to analyse the attributes and relationships of the stakeholders, providing evidence for further policy research on MDR-TB control.

**Methods:**

This study was conducted from October 2018 to March 2019 and applied the stakeholder analysis guidelines and domestic stakeholder analysis. An initial candidate stakeholder list was developed by policy scanning. Ten experts were invited to identify these candidate stakeholders. The major attribute of these stakeholders were analysed using the Michell scoring method. Based on these results, the intertwined relationships among groups of stakeholders were analysed and mapped through a systematic scan of the policy and literature on MDR-TB control, as well as information obtained from the interviews.

**Results:**

A list of 21 types of candidate stakeholders was developed after a literature review and policy scanning, of which 11 received 100% approval. After expert evaluation and identification (the total expert authority was 0.80), 19 categories of stakeholders were approved and included in the stakeholder analysis. We categorized all of the stakeholders into three groups: (i) definitive stakeholders who are mainly involved in administrative departments and the Provincial Center for Disease Control and Prevention (CDC); (ii) expectant stakeholders who are mainly involved with MDR-TB patients, clinical departments of TB hospitals at different levels, community health care facilities, prefectural CDC and charity organizations; and (iii) latent stakeholders who mainly involved family members and neighbours of MDR-TB patients and TB related products manufacturers. Government departments and higher-level CDCs have strong decision-making power in developing MDR-TB control policies whereas the recommendations from service providers and the concerns of patients should be considered.

**Conclusions:**

The MDR-TB prevention system was a multistakeholder cooperation system that was mainly led by government stakeholders. Enhancing communications with front-line service providers and patients on their unmet needs and evidence-based suggestions would highly benefit policy-making of MDR-TB prevention and control.

**Graphical abstract:**

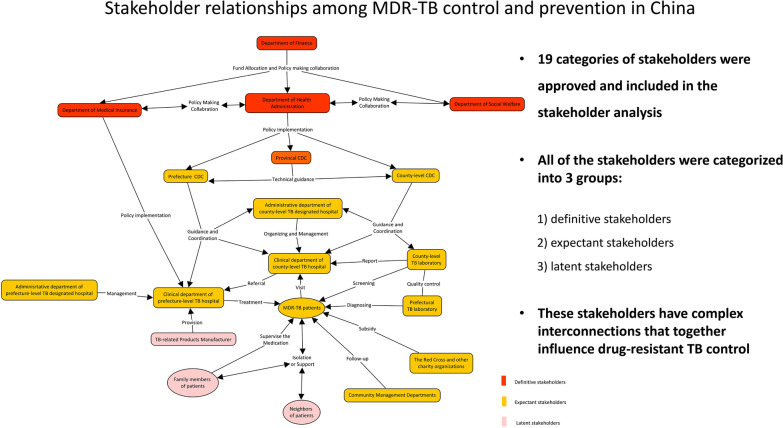

## Background

Multidrug-resistant tuberculosis (MDR-TB) refers to a type of TB that is resistant to two of the most effective first-line drugs: rifampicin and isoniazid [1]. Compared to normal TB, MDR-TB is more difficult to cure and creates a much greater burden to the patients. Because of longer treatment, poorer outcome and lager threat of transmission, MDR-TB is deemed to be a “communicable cancer” [2, 3]. According to a global TB report, MDR-TB continues to be a public health crisis [4]. It was estimated that there were 66 000 MDR-TB/RR (rifampicin-resistant)-TB patients per year in China and the estimated proportions of TB cases with MDR/RR-TB in newly diagnosed and previously treated TB cases were 7.1% and 23% in 2019, respectively [4]. The epidemic of drug-resistant tuberculosis is still a major threat to public health in China [4].

A United Nations (UN) High-Level Meeting on Ending Tuberculosis (TB) was held in New York on 26 September 2018 and stakeholders from different countries and departments attended, aiming to tackle the challenge of TB in the future [5]. In China, the basic TB healthcare unit is set at the county level, where a designated hospital, together with the county CDC, is responsible for the diagnosis, treatment and management of susceptible TB, and the referral of suspected drug-resistant TB patients to up-level (prefectural) designated hospitals. Prefectural designated hospitals are responsible for the diagnosis and treatment of MDR-TB. Different levels of CDC take in charge of policy implementation and disease monitoring on MDR-TB as well. Government departments play roles in policy-making and resource allocation. In TB hospitals/facilities, different departments are involved in the diagnosis and treatment of MDR-TB. It is believed that MDR-TB policy development and implementation could be influenced by various stakeholders; however, who they are, what attributes they have and what roles they play are still unclear in practice.

In reality, government departments coordinate with each other to formulate health policies after identifying the demands from the service objects. Then, the policy is issued by the health administrative department and implemented by the health service facilities. Over the past decade, China has implemented a series of policies on the prevention and control of MDR-TB and established a three-level (province-prefecture-county) prevention and control system [6]. However, most of the current prevention and control strategies were based on experience recommended by the Global Fund to Fight AIDS, TB and Malaria between 2008 and 2013, when the MDR-TB control project was conducted in China [7]. Many challenges have been faced during the process of policy implementation, especially particularly after the completion of the Global Fund project in China. For example, the policy of treatment subsidies and free second-line drug supply for MDR-TB patients could not continue after the project. Drug resistance screening among TB patients has also been suspended in many provinces of China due to financial issues. Consequently, a large number of drug-resistant patients could not be screened and detected. Only approximately half of the diagnosed patients initiated their MDR-TB treatment course, and the cure rate of treatment was less than fifty percent [8]. These problems reflect the poor adaptability and sustainability of current MDR-TB control policies in China.

Stakeholder theory has been widely adopted in health policy research [9]. It reveals that if policy-makers do not consider the interests and influence of important stakeholders during policy development, the result will be setbacks of policy implementation. The definition of “stakeholders” is cited mostly from Freeman, in other words, groups of people who can influence the realization of an organization's goals or all individuals and groups affected by an organization's achievement process of its target [10]. Detecting all of the key stakeholders and understanding their roles and characteristics are important to policy-making and implementation. To understand the role of stakeholders, the "Mitchell Scoring Method", which was proposed by Mitchell, Agger and Wood, is usually applied as an analytical toll [11]. The Mitchell scoring method classifies stakeholders based on three major attributes: power, legitimacy and urgency [12]. Power means, the extent to which the stakeholders have or can gain access to coercive, utilitarian, or normative means to impose their will; legitimacy implies “a desirable social good, that is larger and more shared than merely self-perception and that may be defined and negotiated differently at various level of social organizations”; urgency could be defined as “the degree to which stakeholder claims call for immediate attention” [12]. Different stakeholders could have different involvement of the above three attributes. Stakeholders who are categorized based on their characteristics could impose differently on policy-making and implementation [13]. If the stakeholders have all the three attributes, they could be recognized as the definitive stakeholders. If they had two of three attributes, they could be classified as expectant stakeholder. If there is only one attribute for the stakeholders, they could be latent stakeholders [12].

Because MDR-TB control is mainly conducted at provincial or prefectural levels in China, this study intends to identify key stakeholders during the process of policy-making and implementation for MDR-TB control and prevention at these levels and to analyse their saliences and intertwined relationships of stakeholders, providing a basis for further policy research on MDR-TB control.

## Methods

### Policy scanning and the development of the initial candidate stakeholder list

From October 2018 to March 2019, two researchers (HDB from the hospital medical insurance office and YP from the provincial CDC) searched and collected literature (both in English and Chinese) and policy documents (in Chinese) about MDR-TB prevention and control from the Chinese internet literature database, including the CNKI (China National Knowledge Infrastructure, https://www.cnki.net), Wanfang (http://www.wanfangdata.com.cn/index.html) and Baidu internet search engines (https://www.baidu.com/). Two other researchers (BC and WW from the university and CDC) reviewed policy documents and literature and then summarized the implementation system of MDR-TB control in China. Based on this system, they identified and proposed all possible stakeholder candidates in a list independently from the documents and literature. The two lists were presented to the research group, and those that appeared in both lists were included, while those only in either of the lists were further discussed by the group. After three rounds of group discussion, an agreement was reached, and an initial candidate stakeholder list for MDR-TB control and prevention was composed.

### Expert evaluation and stakeholder identification

Two authors (BC and HDB) designed a stakeholder identification table (supplement document A) based on the candidate stakeholder list, and it was approved by all of the members of the research group. Ten experts working in the national and provincial institutes of MDR-TB programs were invited by Zhejiang Provincial CDC to independently judge whether candidate stakeholders in the lists should be considered to be stakeholders of MDR-TB control and prevention. All of them were willing to take the interview and responded to all of the questions presented in a table. The final inclusion of key stakeholders was determined by the sum of supporting attitudes from the experts. The inclusion criterion was that the proportion of approval given by experts was 70% [14].

### Stakeholder categorization

Based on the results of stakeholder identification, we then used the Michell scoring method to obtain the attributes of the stakeholders which were assessed in three dimensions (power, urgency and legitimacy) on a 1- to 5-point scale [12]. Five points means the highest level of the assessment. We then initiated a second expert survey using an electronic questionnaire that included the scale to inquire 34 experts on MDR-TB control and prevention from national (4), provincial (10), prefectural (10), and county levels (10) about their evaluation of the three attributes of stakeholders. The degree of expert authority was determined by the judgement basis of the indicators (Ca) and familiarity with the indicators (Cs). The experts’ judgement measurement was based on a four-option question (each question has four options to reflect the degree of experience, theoretical analysis, peer understanding, intuition), and familiarity was based on five options (Are you familiar with the field of MDR-TB control? The answer items ranged from 5 to 1, with 5 very familiar and 1 very unfamiliar. The basis score for the judgement was assigned to 0.8/0.6/0.4/0.2, and familiarity was divided into 1/0.75/0.5/0.25/0 [15]. The final degree of expert authoritative coefficient (Cr) was calculated as (Ca + Cs)/2.

We used the median score to represent the stakeholders’ attribute score. If the score was over 3 points on a certain attribute, the corresponding stakeholder should be considered as having this attribute. Based on the methods mentioned above, we finally divided all of the stakeholders into three categories according to the number of attributes they had, i.e., the definitive stakeholder who had all three attributes, the expectant stakeholder who had two of the three attributes and the latent stakeholder, who had only one of the three attributes.

### Relationship analysis and concept mapping

Two of the authors (KL and WW from the CDC) analysed the relationship between every group of stakeholders by systematically scanning the policy and literature on MDR-TB control. Moreover, the relationships within stakeholders were also consulted at the interviews. Two authors (BC and KL) then integrated and mapped all of the key stakeholders based on their attributes (rendered in different colours) and relationships. The stakeholders were linked using lines and arrows based on the understanding of their working roles and relationships in the practice. Then a map was developed to show the intertwined relationship between different types of stakeholders, which was discussed and confirmed by the research group.

**Fig. 1:**
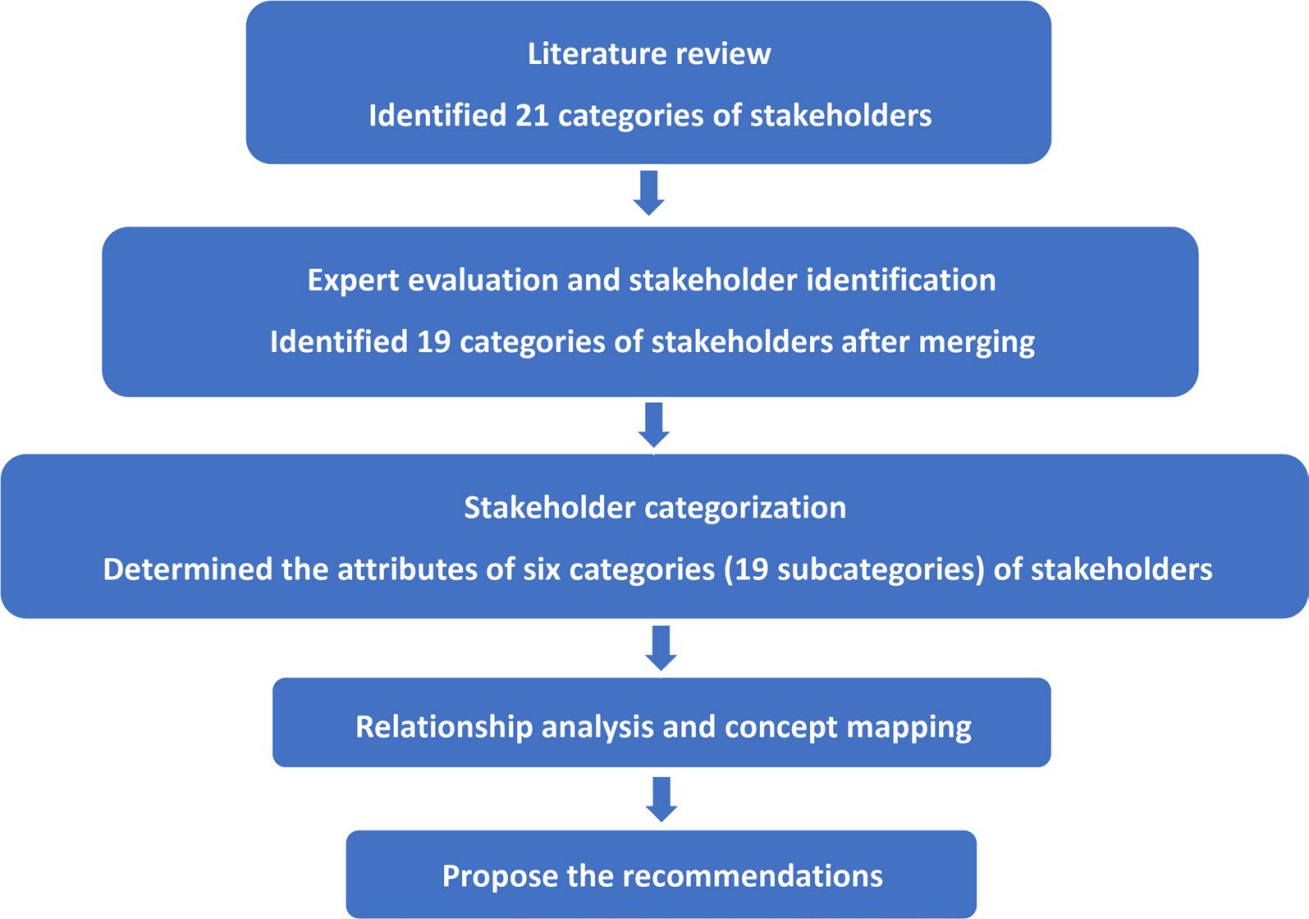
Roadmap of MDR-TB stakeholder identification and classification

The MDR-TB stakeholder identification and classification roadmap is shown in Fig. 1.

### Data analysis

Epidata 3.1 (Jens M. Lauritsen, Odense, Denmark) was used to build a database with double entry and proofreading. The descriptive analysis was performed by Statistical Package for Social Sciences (SPSS) version 18.0 (SPSS Inc., Chicago, Illinois, USA). The median attribute scores of various stakeholders in three dimensions and the degree of expert authority were calculated by the two authors (KL and FW from the CDC) applying SPSS v.18.0. The recording of the interview was sorted into transcripts. Two of the authors (BC and KL) interpreted the answers by the experts and induced major interests and relationships among the stakeholders.

## Results

### Identified stakeholders of MDR-TB prevention and treatment

**Fig. 2:**
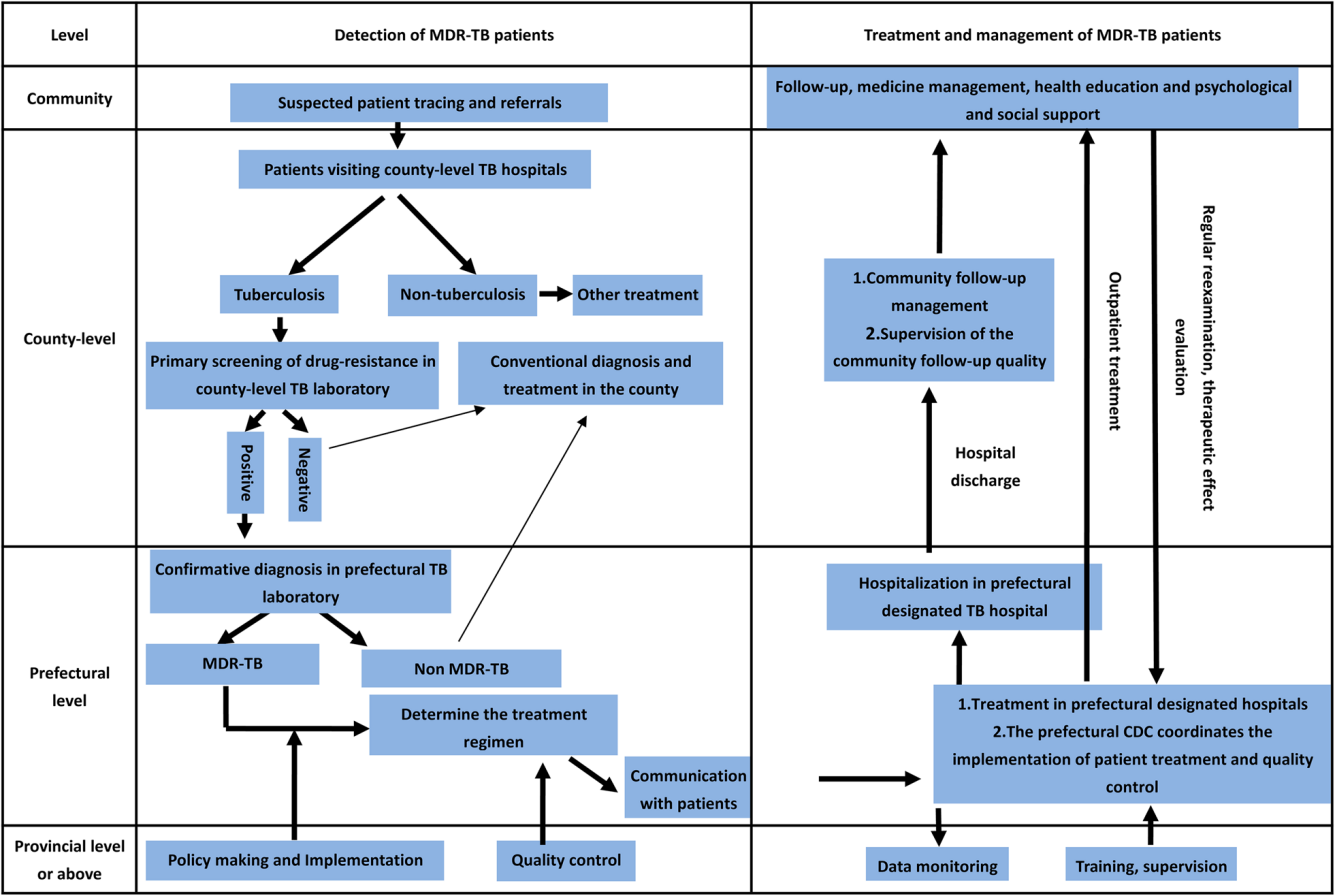
Implementation system of MDR-TB control and prevention in China. *MDR-TB* Multidrug-resistant tuberculosis, *TB* Tuberculosis, *CDC* Centres for disease control and prevention

A total of 152 studies and 120 policy documents related to MDR-TB prevention and treatment were collected and reviewed. Based on current practice and policy documents as well as technical guides for MDR-TB control in China, the implementation system of MDR-TB control and prevention was synthesized, as shown in Fig. 2.

Based on the system and combined with document review, an initial list of 21 types of stakeholder candidates was developed and proposed (Table 1). According to the experts’ opinion, all the candidate stakeholders were approved (at least 70%), with a total of 17 candidate stakeholder categories receiving over 90% approval, including 11 categories receiving 100% approval. 4 candidate stakeholders had approval rates between 80 and 90%. The stakeholder groups of TB drug manufacturers, TB testing reagent manufacturers and TB testing equipment manufacturers were merged into one group called “TB-related products manufacturers”, as suggested by most of the experts. Finally, 19 types of stakeholders came up for the next round of analysis.

**Table 1:** The candidate stakeholder of MDR-TB

Candidate stakeholder	Approval proportion (%)
MDR-TB patients	100.0
Family members of MDR-TB patients	96.7
Neighbours of MDR-TB patients	100.0
Clinical department of county-level TB hospital	100.0
Clinical department of prefectural TB hospital	100.0
Administrative department of county-level TB hospital	100.0
Administrative department of prefectural TB hospital	100.0
County-level TB laboratory	90.0
Prefectural TB laboratory	95.0
Community health care facility	100.0
County-level CDC	100.0
Prefectural CDC	100.0
Provincial CDC	100.0
Department of Health Administration	96.7
Department of Medical Insurance	86.7
Department of Finance	83.3
Department of Social Welfare	83.3
The Red Cross and other charity organizations	80.0
TB Drug manufacturer	100.0
TB Testing reagent manufacturer	90.0
TB Testing equipment manufacturer	90.0

*MDR-TB* Multidrug-resistant tuberculosis, *TB* Tuberculosis, *CDC* Centres for disease control and prevention

### Stakeholder attributes and classification

A total of 34 completed questionnaires were retrieved from expert surveys. All of the questionnaires met the quality criteria and were viewed as valid. The indicator “Ca” was 0.77, and “Cs” was 0.90. The total expert authority was 0.80, which indicates the reliability of the expert evaluation.

The median scores of specific attributes from experts are listed in the following table (Table 2). In line with previous studies, an attribute score greater than 3 is considered to have a corresponding attribute [8]. Following the principle of stakeholder classification, all of the stakeholders were categorized into three groups. Definitive stakeholders were those from the Department of Health Administration, Department of Medical Insurance, Department of Finance, and the Provincial CDC. Expectant stakeholders were MDR-TB patients and providers from the clinical department of prefectural TB hospital, administrative department of county-level TB hospital, county-level CDC, prefectural CDC, clinical department of county-level designated hospital, prefectural TB laboratory, community healthcare facility, county-level TB laboratory, Red Cross and other charity organizations. Latent stakeholders included family members and neighbours of MDR-TB patients and TB-related product manufacturers.

**Table 2:** The median score of the attributes of MDR-TB stakeholders based on the Mitchell scoring method

Attributes of stakeholder	Power	Urgency	Legitimacy
MDR-TB patients	2	3	4
Family members of MDR-TB patients	1	1.5	3.5
Neighbours of MDR-TB patients	1	1	2
Clinical department of county-level TB hospital	2.5	3	4
Clinical department of prefectural TB hospital	3	3	4
Administrative department of county-level TB hospital	3	3	4
Administrative department of prefectural TB hospital	3	3	4
Prefectural level TB laboratory	2.5	2.5	4
County-level TB laboratory	2	2	3
County-level CDC	2	2	4
Prefectural CDC	3	3	4
Provincial CDC	4	4	4
Community health care facility	2	2	4
Department of Health Administration	5	5	5
TB-related products manufacturer	2	2	2
Department of Medical Insurance	5	4	4
Department of Finance	4	4	5
Department of Social Welfare	5	4.5	4
The Red Cross and other charities organizations	3	3	3

*MDR-TB* Multidrug-resistant tuberculosis, *TB* Tuberculosis, *CDC* Centres for disease control and prevention

### Mapping stakeholder relationships in the area of MDR-TB prevention and control

**Fig. 3:**
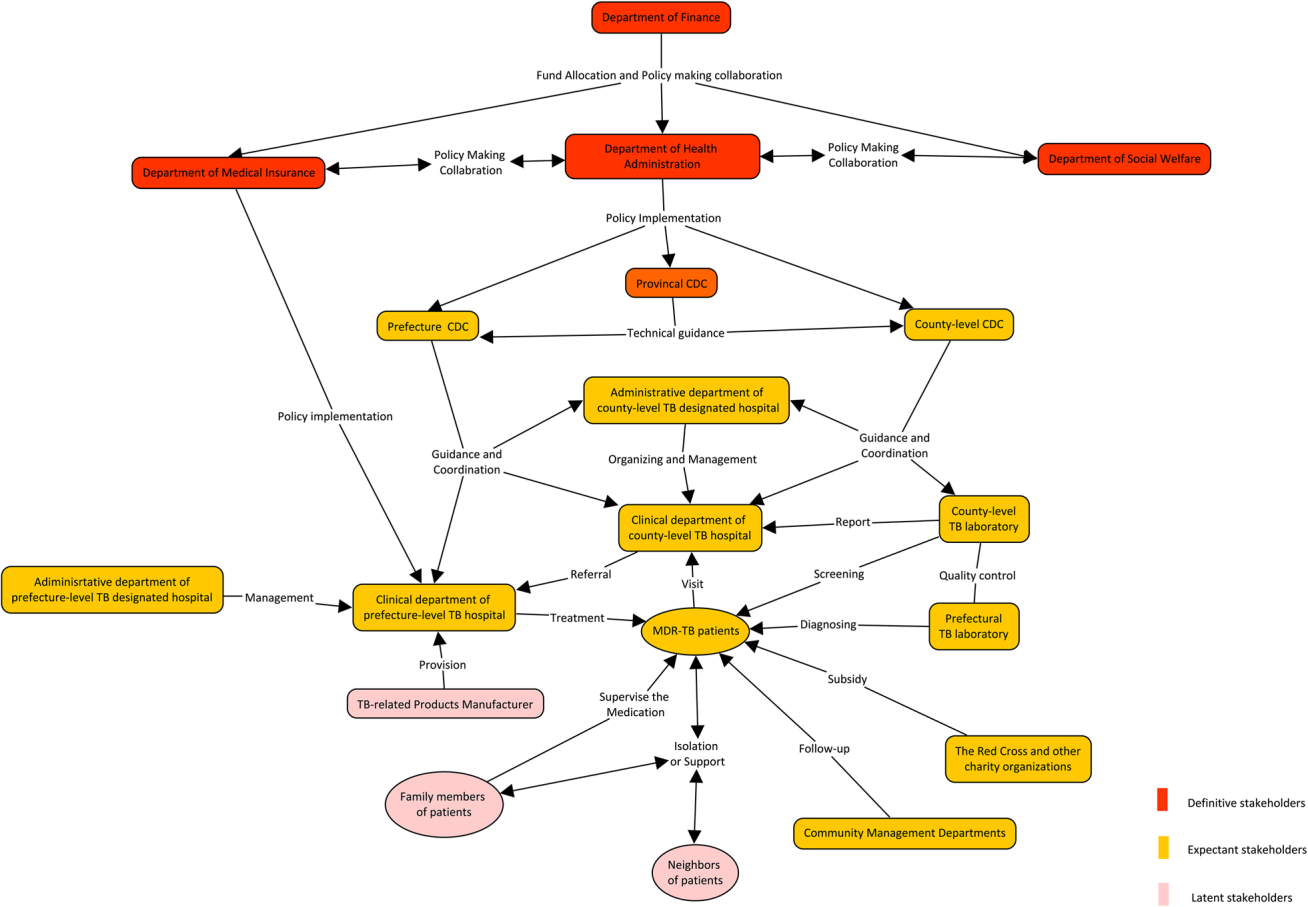
Map of the stakeholder relationships among MDR-TB control and prevention in China. *MDR-TB* Multidrug-resistant tuberculosis, *TB* Tuberculosis, *CDC* Centres for disease control and prevention

As seen from the map (Fig. 3), the colour in red, yellow and pink were applied to represent different categories of stakeholders. Definitive stakeholders such as the Department of Finance, Department of Medical Insurance, Department of Social Welfare, Department of Health Administration and the provincial CDC were at the top of the MDR-TB prevention system. They were considered to be policy makers and planners who coordinated the policy-making process. As policy implementers and practitioners, CDCs at the prefectural and county levels, TB-designated hospitals at all levels and community health centres would play the important roles in the MDR-TB control system. The clinical department and laboratory of TB have taken charge of patient diagnosis and treatment and they also reported case information to CDCs. The administrative department of hospitals should take responsibility for coordination between CDCs and hospitals. All of the CDCs and hospitals were regulated by the local Department of Health Administration. Higher-level CDCs would set technical guides for lower-level CDCs. The Red Cross and other charity organizations provided treatment subsidies and assistance to support MDR-TB patients. Community health centres have been responsible for MDR-TB patient follow-up.

MDR-TB patients should be at the centre of the disease control system, and they have been the service targets of policy. They should be screened at county-level health facilities and referred, diagnosed and finally treated at prefectural TB hospitals. They should also be followed up by community health workers. Their family members and neighbours were potential caregivers and treatment supporters, and they might also monitor the self-managed isolation of patients in the communities. Manufacturers of TB-related products should produce and provide diagnostic equipment and reagents, anti-TB drugs and other necessary products for MDR-TB patients. They did not contact patients directly but had a close relationship with the disease control department and hospitals. The price of the products would ultimately affect the patients, health system and disease control.

## Discussion

The MDR-TB epidemic has been the major challenge of TB control in China. In fact, the controlling work of MDR-TB involves stakeholders not only from medical departments at different levels but also from policy-making departments and non-government organizations. Stakeholder analysis has recently been widely used in the area of public health research, especially in policy research on AIDS and acute infectious diseases [16, 17]. However, there are few relevant studies in the area of TB or MDR-TB control to address low-case detection, limited treatment coverage and unsatisfactory treatment outcomes at present. Previous studies have shown that effective public health policies must meet the interests of most stakeholders [18]. To the best of our knowledge, this study is the first to apply the method of stakeholder analysis to identify the key stakeholders and to explore their attributes in the area of MDR-TB prevention and control. A stakeholder map was also developed based on their working roles and interactions to show the entwined relationships in the prevention and treatment of MDR-TB. These results can provide a reference for future policy-making and research on MDR-TB control in China.

Findings from this study showed that the Provincial CDC and government departments such as the Department of Health Administration, Department of Medical Insurance, Department of Social Welfare and Department of Finance had high scores in all dimensions of attributes, which is similar to the results of other stakeholder research [19], and indicates that these types of stakeholders must have a greater impact on policy development for MDR-TB control. The administrative department, which is at the top of the MDR-TB control system, has more resources and power for resource allocation. Nevertheless, some concerns remain. For example, these stakeholders have limited communication with the grassroots and insufficient information for policy-making, which leads to many problems in policy implementation [20]. Therefore, on the one hand, policy implementing organizations such as CDCs should promptly detect problems in the area and provide evidence-based solutions and suggestions to administrative departments. On the other hand, when administrative departments develop a health-related policy, they should also provide information and discussion time to all the stakeholders from the implementation organization and grassroots.

Our study indicated that among all expectant stakeholders, five types of stakeholders (MDR-TB patients, health providers in the clinical department of county-level designated hospitals, county-level CDCs, community health care facilities, and county-level CDCs) had one or two lower score dimensions, while the legality dimension had a higher score. Except for MDR-TB patients, the other four types of stakeholders are at the frontline of MDR-TB control and are mainly involved in the treatment and community follow-up of patients with MDR-TB playing an important role in disease surveillance and control of MDR-TB [21]. They might have many reasonable demands, while their suggestions have little impact on policy change, thus leading to the result of unsatisfied needs. Moreover, MDR-TB control work has potential risks of infection among these grassroots health workers, which could restrain their working enthusiasm [22]. However, at present, the lack of support of policies and measures for grassroots staff has led to a shortage of human resources at the frontline of TB prevention and control [23]. More attention should be given to the requirements and aspirations of frontline anti-tuberculosis workers during policy making.

In addition, this study showed that stakeholders such as relatives and neighbours of MDR-TB patients, and manufacturers had lower scores in all dimensions. Although they are latent or marginal stakeholders, they also play an important role in MDR-TB control in certain circumstances. The manufacturer who provides drugs, various reagents and medical devices for all patients is indirectly related to patients. The patient's relatives and neighbours, as part of the patient's social relationship, jointly take responsibility for the patient's health education and medication supervision; thus, they also play an essential role in the patient's treatment compliance and the prevention of MDR-TB transmission. Based on current policies, because many expectant and latent stakeholders seldom participated in the process of policy-making, we should strengthen communication with these marginal stakeholders such as primary health workers, patients and manufacturers, in such a way that they would voluntarily participate in the prevention and control of MDR-TB.

With a comprehensive definition and classification of MDR-TB stakeholders, this study proposes recommendations for policy-making for MDR-TB prevention and control. It also has great relevance to the prevention and management of other infectious diseases. With the socialeconomic development, China's major infectious disease prevention system has been much improved [24]. A stakeholder-oriented prevention and control policy-making system for infectious diseases should be established to make continuous progress on the prevention and control of infectious diseases. At the same time, we should enhance communication between the government, CDCs, TB hospitals, doctors, patients, manufacturers and other stakeholders. The interests of all stakeholders must be balanced in such a way that resources can be rationally allocated and the MDR-TB control system can function well.

There are some limitations in our study. First, the inclusion of key stakeholders is determined by the supportive attitudes of the experts and may be influenced by the subjective attitudes of the experts. Second, though the ten experts involved in the decision of the candidate stakeholders were from the national and provincial institutions, they may be lacking in terms of representation.

## Conclusions

The MDR-TB control and prevention system was a multistakeholder cooperation system that was mainly led by government stakeholders. Enhancing the multisectoral cooperation was important for the policy-making process of MDR-TB control. First, the government department should involve more stakeholders in decision making for evidence-based suggestions, especially service providers at different levels. Second, different interests and relationships among stakeholders should be accounted for in the policy-making process. Last but not least, solid measures should be taken to empower more expectant and latent stakeholders, such as front-line service providers and MDR-TB patients, for their voluntary participation in policy-making for MDR-TB control and prevention.

## Data Availability

The datasets used and analyzed during the current study are available from the corresponding author on reasonable request.

## Abbreviations

MDR-TB: Multidrug-resistant tuberculosis
UN: United Nations
CDC: Centre for Disease Control and Prevention
TB: Tuberculosis
RR-TB: Rifampicin-resistant tuberculosis
